# Seasonal human coronavirus NL63 epidemics in children in Guilin, China, reveal the emergence of a new subgenotype of HCoV-NL63

**DOI:** 10.3389/fcimb.2024.1378804

**Published:** 2024-04-26

**Authors:** Renhe Zhu, Rundong Cao, Lulu Wang, Yue Gong, Qian Cheng, Hu Long, Dong Xia, Qinqin Song, Zhiqiang Xia, Mi Liu, HaiJun Du, Juan Song, Jun Han, Chen Gao

**Affiliations:** ^1^ National Key Laboratory of Intelligent Tracking and Forecasting for Infectious Diseases, National Institute for Viral Disease Control and Prevention, Chinese Center for Disease Control and Prevention, Beijing, China; ^2^ Epidemic Prevention and Control Department, Guilin Center for Disease Control and Prevention, Guilin, China

**Keywords:** human coronavirus NL63, new subgenotype, molecular epidemiology, clinical characteristics, whole-genome sequencing

## Abstract

**Introduction:**

Seasonal human coronavirus NL63 (HCoV-NL63) is a frequently encountered virus linked to mild upper respiratory infections. However, its potential to cause more severe or widespread disease remains an area of concern. This study aimed to investigate a rare localized epidemic of HCoV-NL63-induced respiratory infections among pediatric patients in Guilin, China, and to understand the viral subtype distribution and genetic characteristics.

**Methods:**

In this study, 83 pediatric patients hospitalized with acute respiratory infections and positive for HCoV-NL63 were enrolled. Molecular analysis was conducted to identify the viral subgenotypes and to assess genetic variations in the receptor-binding domain of the spiking protein.

**Results:**

Among the 83 HCoV-NL63-positive children, three subgenotypes were identified: C4, C3, and B. Notably, 21 cases exhibited a previously unreported subtype, C4. Analysis of the C4 subtype revealed a unique amino acid mutation (I507L) in the receptor-binding domain of the spiking protein, which was also observed in the previously reported C3 genotype. This mutation may suggest potential increases in viral transmissibility and pathogenicity.

**Discussion:**

The findings of this study highlight the rapid mutation dynamics of HCoV-NL63 and its potential for increased virulence and epidemic transmission. The presence of a unique mutation in the C4 subtype, shared with the C3 genotype, raises concerns about the virus’s evolving nature and its potential public health implications. This research contributes valuable insights into the understanding of HCoV-NL63’s epidemiology and pathogenesis, which is crucial for effective disease prevention and control strategies. Future studies are needed to further investigate the biological significance of the observed mutation and its potential impact on the virus’s transmissibility and pathogenicity.

## 1 Introduction

Acute Respiratory Infection (ARI) poses a significant public health challenge, leading to substantial morbidity and mortality on a global scale (Collaborators GDaI, 2020). Notably, acute lower respiratory infections (ALRI), encompassing pneumonia and bronchiolitis stemming from bacterial and viral sources, stand out as a primary cause of hospitalization and nosocomial deaths among young children, particularly in low- and middle-income countries (Collaborators GI, 2019). In the Chinese context, prevalent viruses in children hospitalized with ARI include respiratory syncytial virus, influenza virus, rhinovirus, human metapneumovirus, adenovirus, parainfluenza virus, bocavirus, and human seasonal coronavirus (Li et al., 2021).

Human seasonal coronavirus NL63 (HCoV-NL63) holds a prominent place among respiratory tract infections. Initially identified in Amsterdam, the Netherlands, in 2004, HCoV-NL63 belongs to the Coronaviridae family and is characterized by its enveloped, single-stranded, positively stranded RNA virus, encompassing 27,553 nucleotides (van der Hoek et al., 2004). Since the emergence of SARS-CoV-2 in late 2019, seven human coronaviruses (HCoVs) have been identified. Remarkably, HCoV-NL63 shares the same cellular receptor, ACE2, for cell entry with SARS-CoV and SARS-CoV-2, distinguishing it from the other three seasonal coronaviruses (HCoV-229E, HCoV-OC43, and HCoV-HKU1) (Lin et al., 2011; Lim et al., 2016). The spike glycoprotein (S) emerges as the primary antigenic protein, housing S1 and S2 domains responsible for viral binding to human angiotensin-converting enzyme 2 (ACE2) and membrane fusion, respectively. Notably, the S1 domain stands out as the most variable gene in the entire HCoV-NL63 genome, serving as a target in molecular epidemiological analyses (Lin et al., 2011).While HCoV-NL63 typically causes mild, self-limiting upper respiratory tract infections across all age groups, it occasionally leads to more severe lower respiratory tract infections, such as pneumonia and bronchiolitis, particularly in susceptible individuals like young children, the elderly, and immunosuppressed patients (Konca et al., 2017; Mayer et al., 2016; Oosterhof et al., 2010; van der Hoek, 2007).

Given the absence of a well-established molecular epidemiological surveillance system for HCoV-NL63 and limited research on its genotypic distribution and genetic characterization, our study aimed to contribute valuable sequence information and in-depth analysis of the molecular evolutionary features of HCoV-NL63, such as nucleotide homology, phylogeny, potential recombination events, N-glycosylation sites, major amino acid substitution sites, etc (Shao et al., 2022). In this study, Nasopharyngeal swabs from children hospitalized with acute respiratory infections were collected and subjected to viral profiling and whole-genome sequence amplification. Subsequent genomic phylogenetic and evolutionary analyses revealed the emergence of a new HCoV-NL63 genotype (type C4) in Guilin, China. This discovery sheds light on the evolutionary trajectory and potential epidemic risks associated with seasonal coronaviruses, offering novel insights for molecular epidemiological studies and control strategies for human coronavirus NL63.

## 2 Materials and methods

### 2.1 Clinical specimens

Nasopharyngeal swab samples from pediatric cases were collected between September 2021 and October 2022 at sentinel hospitals in Guilin, Guangxi Zhuang Autonomous Region. A total of 638 throat swab samples were obtained from children hospitalized for acute respiratory infections (ARI). Following collection, the throat swabs were immediately stored at -80°C and transported in dry ice throughout the cold chain to the laboratory of the Virus Resource Center at the Chinese Center for Disease Control and Prevention for further testing.

Clinical diagnostic criteria for pediatric ARI followed the “Sentinel Monitoring Plan for Severe Acute Respiratory Infection Cases in Hospitals (2011 edition)” issued by the Ministry of Health of the People’s Republic of China (**Supplementary S1**) (China MoHotPsRo, 2011).

Considering the subtropical monsoon climate of Guilin, clinical data for the children were grouped by season as follows: March to May (spring), June to August (summer), September to November (autumn), and December to February of the following year (winter).

### 2.2 Nucleic acids extraction and virus detection

Nucleic acids extraction and virus detection were performed using the GeneRotex96 fully automated nucleic acid extractor (Xi’an Tianlong Technology Co., Ltd.) and the corresponding nucleic acid extraction kit (ZTLJB-Y64T), following the manufacturer’s instructions. The extracted nucleic acids from nasal and pharyngeal swab specimens were subjected to detection for 15 common respiratory viruses using real-time quantitative polymerase chain reaction (qPCR) with the Applied Biosystems QuantStudio 5 instrument. The detected viruses included human rhinovirus (HRV), respiratory syncytial virus (RSV), human metapneumovirus (hMPV), influenza viruses A and B (FluA and FluB), parainfluenza virus types 1-3 (PIV1-3), and human coronaviruses (OC43, NL63, 229E, and HKU1), adenovirus (AdV), human bocavirus (HBoV), and human parvovirus B19 (HPV B19). Primers and probes for these 15 respiratory pathogens were designed in-house, synthesized, and validated by Shanghai Bioengineering Co., Ltd. The GoldStar Probe Mixture kit was used for DNA virus detection, and the GoldStar Probe One Step RT-qPCR Kit was used for RNA virus detection.

### 2.3 S1 domain gene amplification

Positive samples with a Ct value ≤30 from real-time RT-PCR nucleic acid detection of human coronavirus HCoV-NL63 were selected. The TAKARA PrimeScript™ One Step RT-PCR kit Ver. 2 was used for reverse transcription-polymerase chain reaction (RT-PCR) to amplify the nucleotide sequence of the S1 domain. The primer sequences are detailed in **Table 1**. The reaction program included incubation at 50°C for 30 min, initial denaturation at 94°C for 2 min, followed by 35 cycles of denaturation at 94°C for 30 s, annealing at 50°C for 1 min, extension at 72°C for 2 min, and a final extension at 72°C for 10 min. The amplified fragments were sent to Qingke Biosciences Co., Ltd. for Sanger sequencing.

**Table 1:** RT-PCR primer sequences for amplifying the full-length genome and partial Spike gene of HCoV-NL63.

Primer	Sequence (5’-3’)	Target (nt)	Size
**1F**	AATGCTAATCTCTCTATGTTACAATTA	20504	1686
**1R**	GGTGGCTTCAAGTGGAAAATTAC	22189	
**2F**	CGCGTTAAGAGTGGTTCACCAGGTG	22059	1623
**2R**	CAAAGCTGCAAGCCGTCCAGTAATT	23681	
**3F**	TTCAATTCAAGCCGATCAACAAGTT	23624	1600
**3R**	GTCATCAATTAATCGAAGGAACATC	25223	
**4F**	CAACTATACGAAGATGTTCCTTCG	25153	1697
**4R**	CAAAGCACTGAATAACATTTTCCTC	26849	
**5F**	AACCTCGTTGGAAGCGTGTTC	26800	537
**5R**	CTGGCCTACCATTGTGTGTAAG	27553	
**6F**	TGAGGATGTTTGTGTTTGTTTTGAC	18864	1710
**6R**	GTCAGGAACACCTAATTGTAACATA	20573	
**7F**	CTTATGCACACACTTTCTTGTCG	17234	2001
**7R**	ATTTTTCAACACCTTTATCACCCTTA	18915	
**8F**	ATTCAGCAACTGGTTCCTTAGATGT	15479	1808
**8R**	GTTATCGCCACAAACATGAGCACTT	17286	
**9F**	TAATGTCCTCCCTACTATGACAC	14005	1651
**9R**	AAAAGCATAAGAAGACTTAACACTCTC	15600	
**10F**	GACCGTACAACTATTCAAAGTGTTG	12398	1659
**10R**	GTTCTTTACCACTAATAGCATACTT	14056	
**11F**	GGGCTATGGCTAATGGTTATACAAG	9801	1435
**11R**	TTTGCGATATTCATGGCACGCTTCA	11235	
**12F**	GGTCTTGATGGCCTTATTGATTC	11090	1601
**12R**	TTGCTCGTGTTCCATAACCGAC	12467	
**13F**	CAACCACTGTAACTAGCTTTCATGG	7758	2103
**13R**	CTGCCAAAATAGAATAGCACTCAAC	9860	
**14F**	GTCTTCAAAGGGTCAAAAGGGT	5424	2394
**14R**	CGAACACAGTGTAAAGGTGCTT	7817	
**15F**	CAATCTGATAATAATTGTTGGATTAGTA	3424	2043
**15R**	AGATAATGCCTCTTCAGCATCAC	5466	
**16F**	GCAGATGTTCCAGATGCTTTTCAAT	1637	1864
**16R**	GCAACTGTACAAGTGTGGTACTAAT	3500	
**17F**	CAGCAATTATGTTCTTCAGGACTTT	565	1118
**17R**	GTGTAAATGTGCGATAAACTGATTG	1682	
**18F**	TGGTGTTCCGTCACTGCTTATT	1	856
**18R**	AGAAGATAAGTAACCATCCCATGC	1056	
**S1-F**	AGCTGGTAACACTATTCATGCTAAT	20256	2612
**S1-R**	GTAGCACAATCAACAACTATTGGAGT	22843	

1F/R-18F/R whole genome amplification; S1F/R for partial S genes amplification.

### 2.4 HCov-NL63 whole genome amplification

The HCoV-NL63 isolate from the Netherlands (HCoV-NL63_Amsterdam 1, GenBank accession number: NC_005831) served as the reference strain. Eighteen primer pairs covering overlapping regions were designed using the Blast-Primer web tool. RT-PCR was employed to amplify specific segments with overlapping regions, encompassing all protein-coding genes except the 5’ untranslated regions (UTR) and 3’ UTR. Primer pairs and amplification details are provided in **Table 1**. Samples with Ct values ≤25 from human coronavirus HCoV-NL63 real-time RT-PCR were selected, and the TAKARA PrimeScriptTM One Step RT-PCR kit Ver. 2 was used for RT-PCR amplification. The amplified segments were subjected to Sanger sequencing by Qingke Biosciences Co., Ltd.

### 2.5 Reorganization analysis

SimPlot 3.5.1.4 software was used for intraspecific whole-genome recombination analysis of the 13 HCoV-NL63 sequences obtained in this study against the reference sequence (Lole et al., 1999). A sliding window size of 400 bp with a step size of 40 bp was employed.

### 2.6 Phylogenetic analysis

As of October 11, 2023, all HCoV-NL63 S gene sequences were retrieved from NCBI to establish a dataset. Strain sequences with incomplete, excessively ambiguous bases, and unclear sampling years or countries were excluded. For identical sequences from the same region and year, only one was retained. The nucleic acid sequence alignment was performed using MAFFT software (Katoh et al., 2019). To confirm the completeness of the time signal of the HCoV-NL63 complete S gene database, IQ-TREE was used to build a maximum likelihood phylogenetic tree (ML-TREE). The phylogenetic tree utilized a non-molecular clock with 1000 repetitions of the best-fitting model. TempEst v1.5.3 was used to visualize a root-to-tip regression plot based on regression analysis.

Time-scaled phylogenetic tree inference for the complete HCoV-NL63 S gene sequence dataset with a time signal was performed using the Bayesian Markov Chain Monte Carlo (MCMC) method in BEAST v.1.10.4.3. The optimal nucleotide substitution model recommended by ModelFinder was selected based on Bayesian scores. The evolutionary model comprised TN93+Empirical+I+four gamma categories. The molecular clock model selected a log-normal distribution of uncorrelated relaxed clock (UCLN) based on previous references, and the tree prior model chose Bayesian SkyGrid. The time to the most recent common ancestor (tMRCA) was set at twice the height of the tree, with other parameters using default settings from BEAUti. The MCMC chain length was set to 200,000,000, with sampling every 20,000 steps. The XML file created in BEAUti v1.10.4 was run in BEAST, and the generated log file was loaded into Tracer v1.10.4 to confirm that all parameter estimates had effective sample sizes (ESS) exceeding 200, indicating sufficient convergence. The TreeAnnotator program in the BEAST package was used to construct the maximum clade credibility tree (MCC-tree) with a 10% burn-in, and FigTree v1.10.4 was employed to visualize the MCC-tree.

For the full-length coding sequence of HCoV-NL63, ORF1ab, and S1 domain, a neighbor-joining phylogenetic tree was constructed using MEGA v7.0 (Kumar et al., 2016) software with 1000 bootstrap repetitions and the Kimura2‐parameter nucleotide substitution model.

### 2.7 Amino acid variant sites analysis

Amino acid variation characteristics were analyzed based on the Spike protein amino acid sequence of the representative strain of HCoV-NL63. Amsterdam I strain (GenBank accession number: NC 005831) and HCoV-NL strain (GenBank accession number: AY518894) were chosen as reference strains for A and B genotypes, respectively.

### 2.8 Statistical analysis

Data organization and statistical analysis were conducted using Excel 2021 and SPSS 26.0 software. Differences in rates (or composition ratios) between groups were compared using the χ^2^ test or Fisher’s exact probability method. A two-sided test was applied with a significance level of α=0.05 (*P*<0.05).

## 3 Results

### 3.1 Clinical data of HCoV-NL63 infected patients

From 2021 to 2022, a total of 638 children hospitalized in Guilin, Guangxi Zhuang Autonomous Region, due to acute respiratory infections, were tested for HCoV-NL63. Among them, 83 cases tested positive for HCoV-NL63 (**Table 2**). Although the detection rate of seasonal coronaviruses in Guilin for the same period in previous years is lacking, the detection rate of 13.01% (83/638) is significantly higher than the national average detection level of four common seasonal HCoVs from 2009 to 2019 (Li et al., 2021). Considering the sharp increase in cases from July to September and the composition ratio of single infections, it suggests a localized outbreak of HCoV-NL63 in Guilin in 2022, which is relatively uncommon.

**Table 2:** Positive rate and proportion of four seasonal coronaviruses in ARI cases.

Virus	Positive cases	Positive rate (%)	Proportion (%)
**Single infection**	284	44.51	77.81
NL63	45	7.05	12.32
OC43	6	0.94	1.64
229E	0	0	0
HKU1	0	0	0
**Double infections**	70	10.97	19.18
NL63+RSV	15	2.35	4.11
NL63+AdV	7	1.1	1.92
NL63+HBOV	6	0.94	1.64
NL63+HRV	3	0.47	0.82
OC43+HRV	1	0.16	0.27
OC43+FluB	1	0.16	0.27
OC43+HBOV	1	0.16	0.27
**Multiple infections**	**12**	**1.88**	**3.29**
NL63+AdV+RSV	3	0.47	0.82
NL63+HBOV+FluA	1	0.16	0.27
NL63+HRV+FluA	2	0.31	0.55
NL63+AdV+RSV+FluB	1	0.16	0.27

The bold values represents the number and percentage of patients infected with HCoV-NL63 in this study.

Most patients with HCoV-NL63 infection presented with upper respiratory tract infections, while 5 cases had pneumonia, 6 cases had bronchitis, and 2 cases had acute gastroenteritis. The age of the children ranged from 2 months to 12 years, and there was no statistically significant difference in detection rates between age groups and gender groups. Infections showed clear seasonality, with the peak of positive cases in August (**Supplementary S2**). The clinical information of HCoV-NL63-infected children is summarized in **Table 2**. The clinical manifestations included cough, fever, nasal congestion, shortness of breath, wheezing, abnormal lung breath sounds, and diarrhea. No significant statistical difference was observed in clinical presentation and diagnosis among different types of HCoV-NL63, including C3, C4, and B types (see **Supplementary S3**). Most children exhibited mild, self-limiting upper respiratory tract infections, with a small number experiencing gastrointestinal symptoms, consistent with previous reports (Kiyuka et al., 2018; Wen et al., 2022; Huang et al., 2017).

### 3.2 Co-infections

In addition to HCoV-NL63, samples from positive patients were tested for 14 other common respiratory viruses (**Supplementary S4**). **Table 3** presents the detection results of the four seasonal coronaviruses. Among the 83 patients, 45 cases (54.22%) had HCoV-NL63 as a single infection, while 38 cases (45.78%) had co-infections with other viruses such as RSV, HBoV, and HRV (**Table 3**). Clinical characteristics, including age, gender, co-infection, and clinical symptoms, showed no significant differences among different genotypic infections. These data indicate that HCoV-NL63 primarily causes upper respiratory tract infections, and the prevalent strain in Guilin in this study is consistent with pathogenicity reported in previous studies (Zeng et al., 2018; Ye et al., 2023). However, the significantly increased infectivity suggests that the detected HCoV-NL63 epidemic strain may be a human respiratory pathogen with greater pathogenic potential than previously believed.

**Table 3:** Clinical data of children with acute respiratory infections caused by HCoV-NL63.

Clinical information			*χ* ^2^ value	*P* value
Gender			0.002	0.966
Male	49	59.04%		
Female	34	40.96%		
Age			0.317	0.854
< 2 years	38	45.78		
2-5 years	28	33.73		
> 5 years	17	20.48		
Diagnosis				
Pneumonia	5	6.02%		
Acute bronchitis	6	7.23%		
Acute upper respiratory tract infection	70	84.34%		
Gastroenteritis	2	2.41%		
Co-infection				
RSV	15	18.07%		
ADV	7	8.43%		
HBOV	6	7.23%		
HRV	3	3.61%		
ADV+RSV	3	3.61%		
HBOV+FLUA	1	1.20%		
HRV+FLUA	2	2.41%		
ADV+RSV+FLUB	1	1.20%		
Single infection	45	54.22%		

### 3.3 Topology of the evolutionary tree reveals the emergence of new HCoV-NL63 genotypic subtypes

A total of 35 S1 domain genes and 13 HCoV-NL63 full-length genomes were obtained. To understand the genetic relationships between the prevalent HCoV-NL63 strain detected in this study and other HCoV-NL63 strains in GenBank, phylogenetic analyses were conducted based on the Spike gene, S1 domain gene, ORF1ab, and full genome sequences.

**Figure 1:**
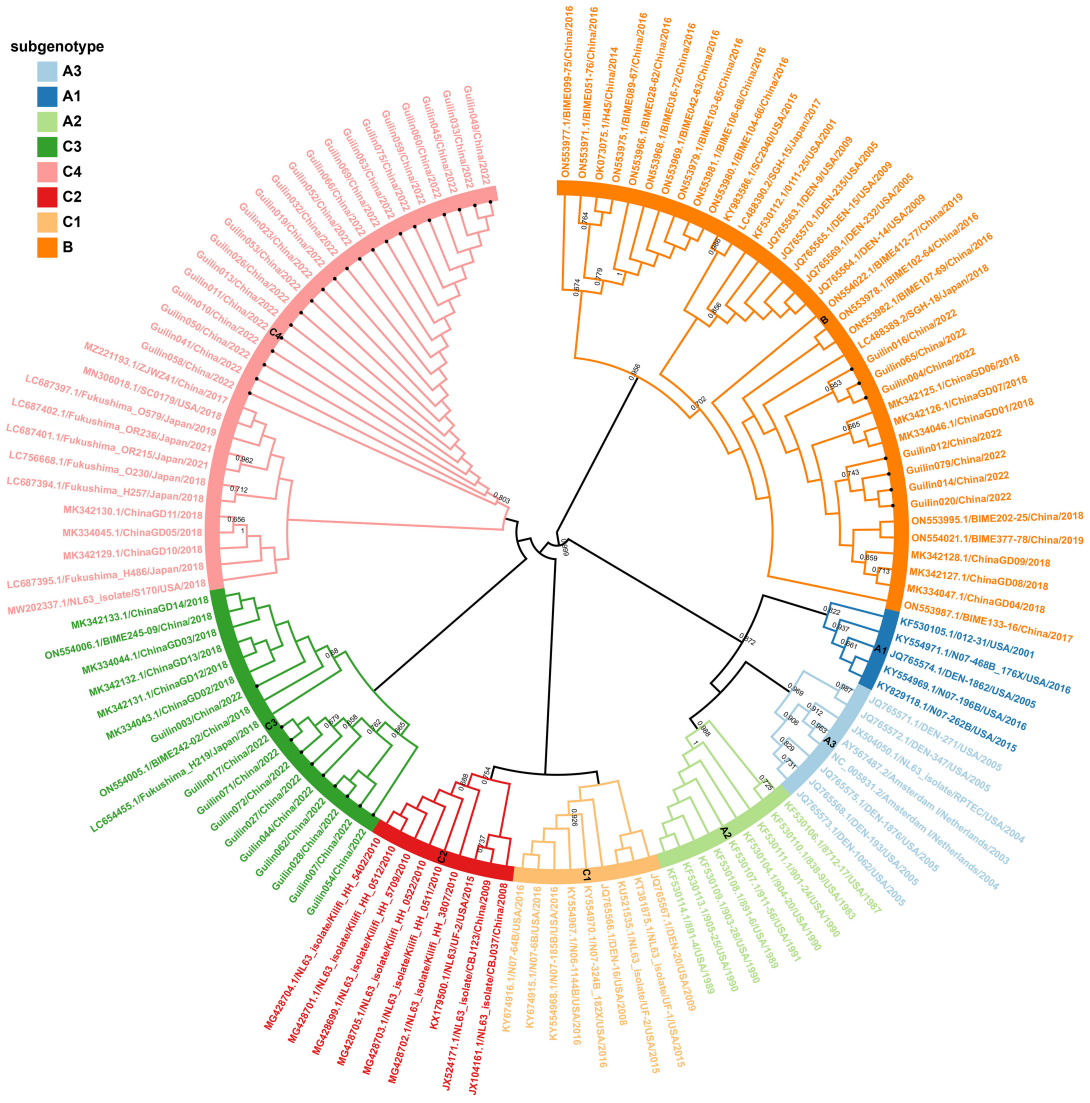
Phylogenetic analyses based on partial spike genes of HCoV-NL63 identified in this study. 38 HCoV-NL63 spike gene partial sequences detected in this study were used for phylogenetic analysis by MEGA 7.0 software using Neighbor-joining method and confirmed the presence of new subgenotype of HCoV-NL63. Bootstrap values greater than 65% were considered statistically significant for grouping. The sequence number in the tree with a circular symbol is the sequence obtained in this study.

In the ARI hospitalized children’s cases in Guilin, both B and C genotypes of HCoV-NL63 were co-circulating, with C being the predominant genotype. Among the 35 HCoV-NL63 S1 domain sequences in this study, they clearly evolved into an independent cluster within the C subtype in the phylogenetic tree. This cluster occupied a relatively distinct position in the phylogenetic tree, different from known C1-C3 genotypic subtypes, and formed a unique lineage, tentatively named as C4 (**Figure 1**). The evolutionary tree showed that the HCoV-NL63 S1 domain gene sequences detected in Guilin exhibited both temporal and spatial clustering, confirming their common ancestry and continuous circulation. Additionally, 12 epidemic strains from Japan, the United States, and other parts of China belonged to the same lineage, indicating that this new lineage may have already spread widely worldwide. The discovery of the new lineage C4 suggests potential risks associated with the rapid mutation of HCoV-NL63, leading to an epidemic of acute respiratory infections. Notably, C4 shared a common origin with the C3 genotypic subtype.

**Figure 2:**
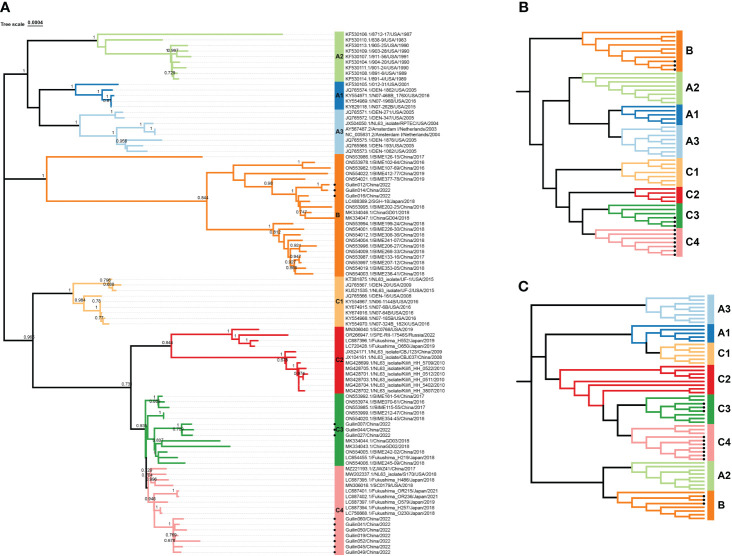
Phylogenetic analysis based on complete genome, S and ORF1ab genes of HCoV-NL63. All available HCoV-NL63 complete genomes from GenBank were collected and used for the evolutional analysis using MEGA 7.0 with 1000 bootstrap replications, Bootstrap values greater than 70% were considered statistically significant for grouping. **(A)**. Five complete genomes derived from this study were in red. Nucleotide sequence alignments were created using MAFFT. Corresponding spike **(B)** and orf1ab **(C)** genes were used for the genotype identification. The sequence number in the tree with a circular symbol is the sequence obtained in this study.

For further evidence supporting this new lineage, phylogenetic analyses were conducted based on the full genome, ORF1ab, and Spike genes, showing similar topologies among the eight genotypic subtypes (**Figures 2A–C**). Systematic phylogenetic analysis further confirmed the emergence of the new lineage C4 of HCoV-NL63 in Guilin, China, suggesting its potential association with the increased cases of acute respiratory infections in 2022. Moreover, some epidemic strains obtained in this study belonged to the new genotypic subtype C3, discovered in 2018, as well as the B genotype. The shared ancestry between the new genotypic subtypes C4 and C3 in the evolutionary structure indicates a common origin. The discovery of the new genotypic subtype C4 suggests that HCoV-NL63 is undergoing continuous mutation, which could potentially contribute to the ongoing outbreak in Guilin.

### 3.4 Phylogenetic analysis of temporal signal analysis and time scale estimation

**Figure 3:**
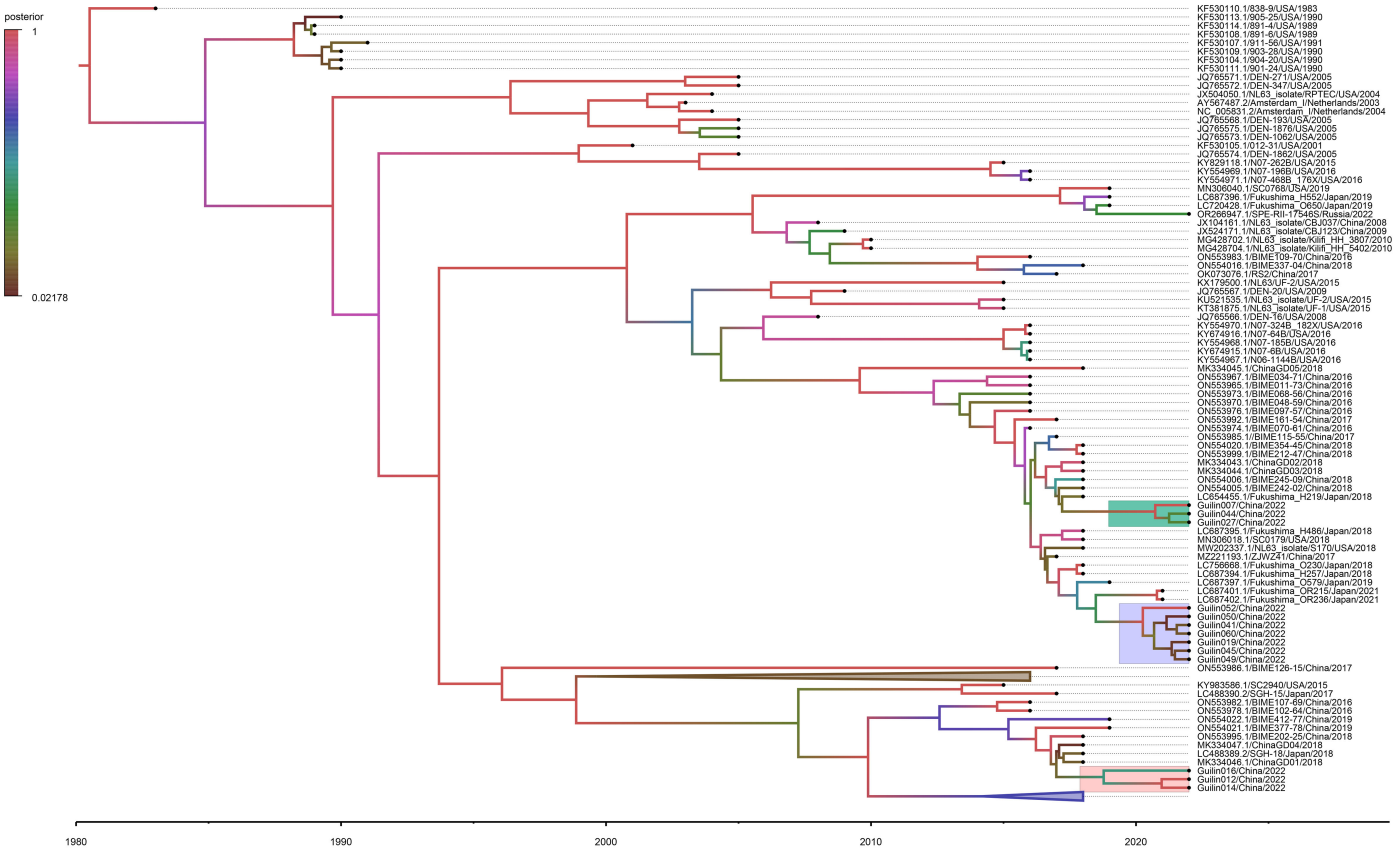
Time-resolved phylogenetic analysis of spike gene of HCoV-NL63 strains. The node label represents the posterior probability. The C4, C3, and B sub-genotype strains detected in the Guilin area are indicated in green, purple, and gray. BEAST software was used to estimate the nearest common ancestor (tMRCA) of the new subgenotype circulating in the Guilin area based on the nucleotide sequence of the Spike gene. Analyses were conducted under the best-fit nucleotide substitution model (TN93+F+I+G4) and using a relaxed (uncorrelated lognormal) molecular clock model.

The Bayesian Evolutionary Analysis by Sampling Trees (BEAST) program is extensively utilized for exploring the spatiotemporal and evolutionary dynamics of viral pathogens through time-stamped nucleotide sequence datasets. Under the non-strict molecular clock model, the Bayesian MCC tree based on the HCoV-NL63 S gene sequence dataset is shown in **Figure 3**. The estimated evolution rate (4.37×10^-4^ substitutions per site per year) and TMRCA date (1978.674) results reveal ESS values exceeding 200, consistent with the results of the time signal analysis (**Supplementary Figure 2**). The MCC tree illustrates that the obtained 13 HCoV-NL63 strains belong to genotypic subtypes C3, C4, and B. They exhibit a certain temporal and spatial clustering, with subtype C4 being closely related to epidemic strains from Japan and the United States. This further confirms the results of the phylogenetic analysis based on the S1 domain, indicating the emergence of a new lineage of HCoV-NL63.

According to the MCC time-calibrated tree estimation, although C3 and C4 share a common ancestor, they diverged around 2015, indicating different evolutionary paths. Overall, the results of phylogenetic analysis suggest that, in recent years (2021-2022), genotypic subtypes C3, C4, and B dominate among all prevalent HCoV-NL63 strains in the Guilin region. This pattern aligns with the predominant circulation of genotypic subtypes C and B of HCoV-NL63 in China.

### 3.5 Reorganization analysis

**Figure 4:**
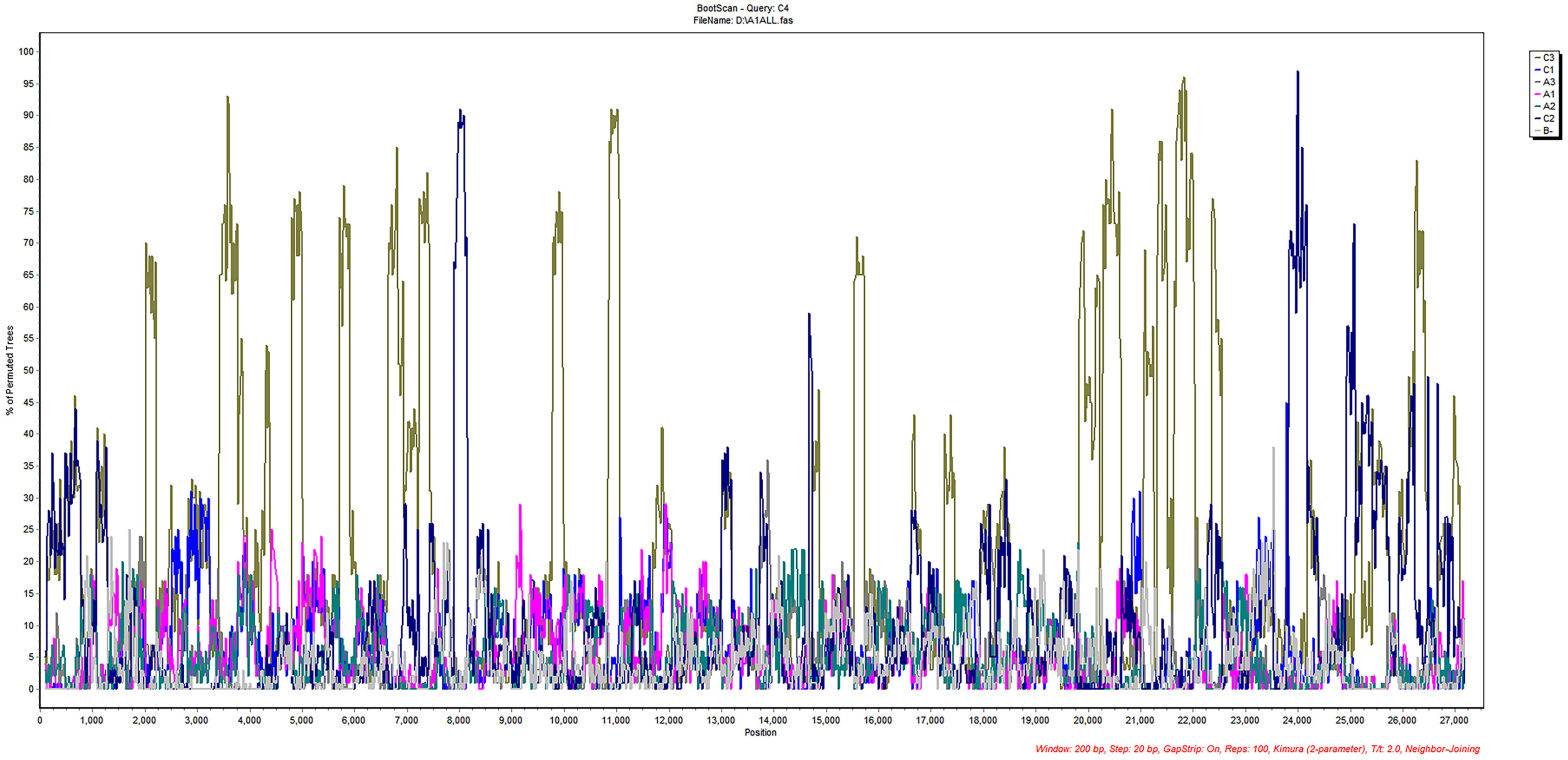
Recombination analyses of HCoV-NL63.Similarity plot and bootscan analysis were performed intraspecies. No obvious recombination events were detected in new subgenotype C4 using Simplot software analysis.

The 13 aligned sequences of HCoV-NL63 were imported into SimPlot software for subsequent recombination analysis. The results of the recombination analysis for HCoV-NL63, as shown in **Figure 4**, indicate no clear potential recombination regions in ORF1ab, S to N. Combining phylogenetic and recombination analyses, it can be suggested that the new genotypic subtype C4 of HCoV-NL63 may result from gene mutations rather than homologous gene recombination. No apparent intraspecific recombination signals were detected, suggesting that the new genotypic subtype C4 of HCoV-NL63 may have originated from gene mutations rather than intraspecific gene recombination.

### 3.6 Amino acid mutations

**Figure 5:**
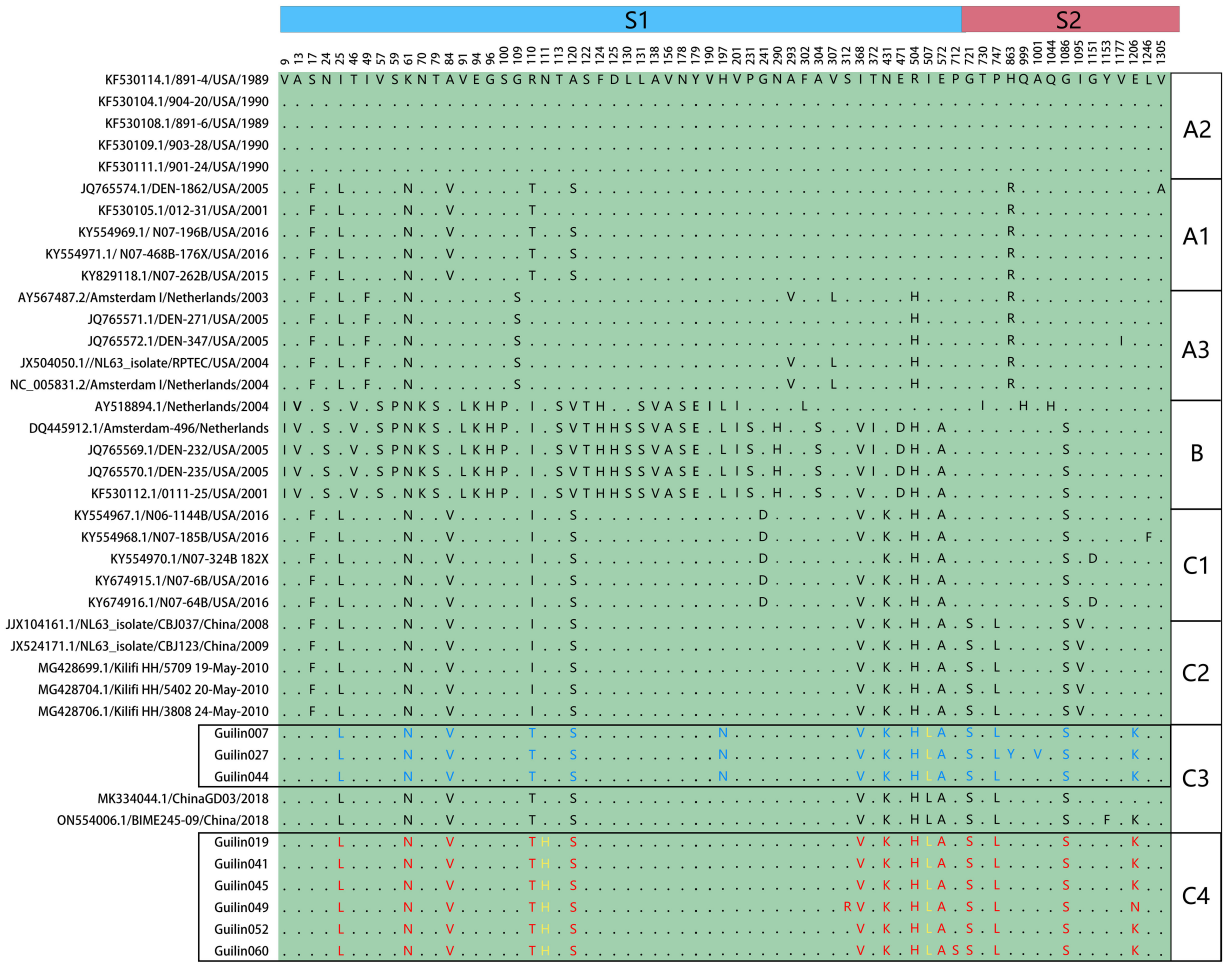
Single amino acid polymorphism analysis of HCoV-NL63 spike protein. All available HCoV-NL63 complete genomes were aligned, and corresponding spike proteins were retrieved and used for single amino acid polymorphism analysis. One unique mutation N111H was identified in yellow.

The spike protein plays a crucial role in the entry and pathogenic mechanisms of coronaviruses. Specific mutations in the amino acid residues of the spike protein, particularly in the receptor-binding domain (RBD) responsible for specific binding to the ACE2 receptor protein, may alter the interaction levels between HCoV-NL63 and host cells, thereby affecting the virus’s virulence or transmission within the population. To further describe the antigenic features of the prevalent HCoV-NL63 strains detected in this study and understand the reasons for the localized outbreak in Guilin, a single amino acid polymorphism analysis (SAP) was conducted on the spike protein sequences, as shown in **Figure 5**.

The SAP analysis identified 65 polymorphic sites in the spike protein, highlighting genotypic-specific SAPs and confirming the rationale behind the new genotypic subtype classification (A1, A2, A3, B, C1, C2, C3, and C4). The amino acid mutation analysis revealed that the C4 subtype in the spike protein had a unique mutation, S574L, at the 574th amino acid residue in the receptor-binding domain (RBD), which is also detected in other C4 subtype epidemic strains in the N-terminal domain (NTD) of the spike protein. Furthermore, a specific mutation, I507L, associated with increased infection capability, was observed in the RBD of the central region of the spike protein in the C4 subtype, belonging to the C3 genotypic subtype. This mutation might be related to the increased number of acute respiratory infections caused by HCoV-NL63 in Guilin in 2022. These findings confirm the validity of the new genotypic subtype classification (A1, A2, A3, B, C1, C2, C3, and C4) and suggest an increased pathogenic risk of HCoV-NL63 with evolution.

## 4 Discussion

Until now, three highly pathogenic human coronaviruses have been identified: SARS-CoV, MERS-CoV, and SARS-CoV-2. Coronaviruses have caused two large-scale public health crises in the past 20 years. As of 2021, the global COVID-19 pandemic caused by Severe Acute Respiratory Syndrome Coronavirus 2 (SARS-CoV-2) has resulted in approximately 6.9 million deaths, posing unprecedented challenges to global health (Drosten et al., 2003; Zaki et al., 2012; World Health Organization (2023)). As mentioned earlier, SARS-CoV, SARS-CoV-2, and HCoV-NL63 share the same receptor, angiotensin-converting enzyme (ACE)-2, when entering host cells. However, the consequences of virus invasion differ significantly. While SARS-CoV and SARS-CoV-2 can cause severe respiratory distress, HCoV-NL63 typically leads to mild respiratory tract infections limited to the upper respiratory tract in most individuals. This may be attributed to the lower affinity of NL63 spike protein for the receptor compared to SARS-CoV-2. Consequently, outbreaks of respiratory infections caused by HCoV-NL63 in children are rarely reported (Nyaguthii et al., 2021; Hand et al., 2018).

In this study, focusing on pediatric patients hospitalized in Guilin, Guangxi Zhuang Autonomous Region, due to acute respiratory infections in 2021-2022, we obtained a total of 13 nearly full-length HCoV-NL63 genome sequences and 25 S1 domain gene sequences. Molecular characteristics, including phylogenetic analysis, time signals, potential recombination events, and amino acid mutations, were described. Compared to national HCoV infection levels in the past 10 years, the number of HCoV-NL63 infection cases in Guilin significantly increased in 2021-2022, indicating a rare small-scale outbreak of acute respiratory infections caused by HCoV-NL63 in Guilin, China. Additionally, phylogenetic and recombination analyses suggested an association between this outbreak and three HCoV-NL63 subtypes (C3, C4, and B), with the identification of a new genetic subtype, C4. C4, homologous to C3, exhibited distinct evolutionary trends, and genetic variations may help the virus adapt to external environments to maintain prevalence in populations.

The spike protein plays a crucial role in coronavirus invasion of host cells and the resulting host disease process. Mutations in the amino acid residues of the spike protein, particularly in the receptor-binding domain (RBD) responsible for specific binding to the ACE2 receptor protein, may alter the interaction levels between HCoV-NL63 and host cells, affecting the virus’s virulence or transmission (Lin et al., 2011; Wu et al., 2009). The study identified specific mutations, such as I507L, in the RBD, which have been confirmed to enhance viral entry (Wang et al., 2020). This may be a contributing factor to the increased infectivity of the HCoV-NL63 C4 genetic subtype in the Guilin region. Furthermore, mutations in the N-terminal domain (NTD) of the spike protein, particularly at N111 and N119, were observed in C4, involving amino acid substitutions R110T and N111H. According to previous reports, the N-linked glycosylation sites at aa positions N111 and N119 made direct polar contacts, and they were linked to aa substitution sites R110I and A120S (Shao et al., 2022). While the importance of these mutations in the NTD region for SARS-CoV-2 has been reported (Cui et al., 2022), further evidence is needed to determine potential changes in spike protein antigenicity resulting from mutations in the N-glycosylation sites of the C4 genetic subtype. In summary, subtype-specific mutations in the spike protein further confirm the rationale behind the new subtype classification (A1, A2, A3, B, C1, C2, C3, and C4). It is important to emphasize that the emergence of the new subgenotype C4 is a natural consequence of virus evolution. Sustained genetic variation aids the virus in adapting to its external environment, thereby maintaining its prevalence in the population-a common and expected phenomenon among RNA viruses. The conclusions drawn in this study are based on limited datasets from specific geographic regions and time periods. Thus, it is premature to draw comprehensive conclusions regarding the global significance of C4 gene subtypes or their associated mutations.

HCoV-NL63 is generally highly susceptible to the human population, with most individuals experiencing their first infection in childhood and neutralizing antibodies against HCoV-NL63 are common in adult serum (Hofmann et al., 2005), but this protective immunity is not durable (Amoretti et al., 2002), leading to recurrent infections. Additionally, like other coronaviruses, HCoV-NL63 may be a zoonotic pathogen originating from bats (Huynh et al., 2012). Interspecies genetic recombination resulting from different HCoV strains carried by bats is one reason for the seasonal/recurrent infection pattern of HCoV-NL63 (Tao et al., 2017). Further research is necessary to elucidate the significance of the unique amino acid N111H mutation in the C4 subtype NTD as observed in this study.

While pathogenic changes are inevitable with the virus’s continuous spread and evolution, considering that HCoV-NL63 and SARS-CoV-2 share the same cell receptor, and given the severe consequences of the global spread of SARS-CoV-2, the potential risks of increased virulence or epidemic spread due to ongoing mutations of HCoV-NL63 are significantly elevated. Therefore, regular epidemiological and phylogenetic studies and the prompt establishment of a comprehensive HCoV-NL63 molecular epidemiological surveillance system are of particular practical importance.

## Data availability statement

The original contributions presented in the study are included in the article/**Supplementary Materials**, further inquiries can be directed to the corresponding author/s.

## Ethics statement

The studies involving humans were approved by Ethics Review Committee of National Institute for Viral Control and Prevention, Chinese Center for Disease Control and Prevention. The studies were conducted in accordance with the local legislation and institutional requirements. Written informed consent for participation in this study was provided by the participants’ legal guardians/next of kin.

## Author contributions

RZ: Writing – original draft, Conceptualization, Data curation, Formal analysis, Methodology, Visualization, Investigation, Software. RC: Investigation, Methodology, Software, Validation, Writing – original draft. LW: Conceptualization, Investigation, Software, Writing – original draft. YG: Formal analysis, Investigation, Methodology, Software, Writing – original draft. QC: Investigation, Methodology, Software, Visualization, Writing – original draft. HL: Investigation, Resources, Supervision, Writing – review & editing. DX: Investigation, Methodology, Supervision, Writing – original draft. QS: Investigation, Methodology, Resources, Supervision, Writing – original draft. ZX: Investigation, Methodology, Supervision, Writing – original draft. ML: Formal analysis, Investigation, Methodology, Supervision, Writing – original draft. HD: Investigation, Software, Supervision, Writing – review & editing. JS: Conceptualization, Investigation, Methodology, Supervision, Writing – review & editing. JH: Formal analysis, Funding acquisition, Methodology, Resources, Supervision, Writing – review & editing. CG: Formal analysis, Investigation, Methodology, Resources, Supervision, Validation, Visualization, Writing – review & editing.
