# Supplementary material for: Seasonal human coronavirus NL63 epidemics in children in Guilin, China, reveal the emergence of a new subgenotype of HCoV-NL63
Source: Front Cell Infect Microbiol. 2024 Apr 26;14:1378804. doi: 10.3389/fcimb.2024.1378804 (PMC11082418; doi:10.3389/fcimb.2024.1378804)
Supplement: Supplementary file 1 [file Image_1.pdf]

S1

Case definition for monitoring severe acute respiratory infections in hospitalized patients:

Hospitalized patients or newly admitted patients aged over 5 years old with the following four clinical manifestations:

(1) acute onset;

(2) axillary temperature ≥38℃;

(3) cough or sore throat;

(4) tachypnea (respiratory rate ≥25 breaths/minute) or dyspnea.

Hospitalized patients or newly admitted patients aged 5 years old and below with the following three clinical manifestations:

(1) acute onset;

(2) cough or dyspnea;

(3) accompanied by one of the following symptoms or signs: ① tachypnea: respiratory rate greater than 60 breaths/minute (<2 months old infants); respiratory rate greater than 50 breaths/minute (2-11 months old infants); respiratory rate greater than 40 breaths/minute (1-5 years old); ② refusal to eat or choking on milk; ③ severe vomiting; ④ convulsions; ⑤ lethargy or coma; ⑥ chest wall retraction or wheezing at rest.
