# Supplementary material for: Seasonal human coronavirus NL63 epidemics in children in Guilin, China, reveal the emergence of a new subgenotype of HCoV-NL63
Source: Front Cell Infect Microbiol. 2024 Apr 26;14:1378804. doi: 10.3389/fcimb.2024.1378804 (PMC11082418; doi:10.3389/fcimb.2024.1378804)

**S2**

Case number and positive rate of HCoV-NL63 infection by month in the children hospitalized for acute respiratory infections in 2022

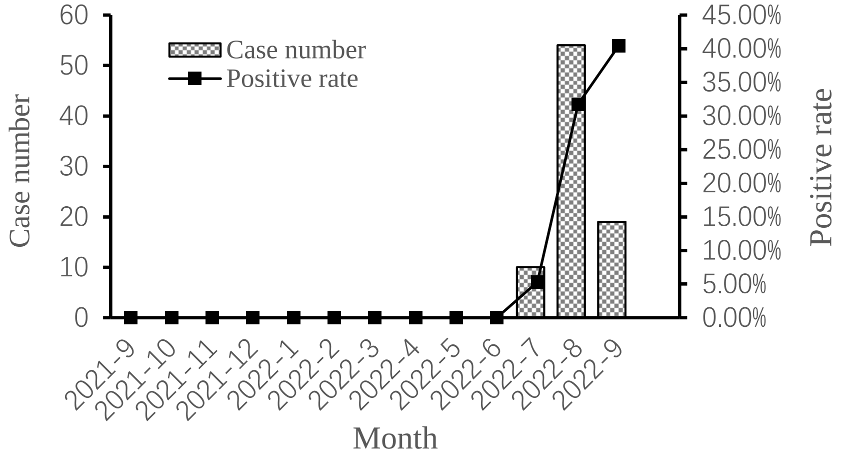

Supplement: Supplementary file 2 [file Image_2.pdf]
