# Supplementary material for: Seasonal human coronavirus NL63 epidemics in children in Guilin, China, reveal the emergence of a new subgenotype of HCoV-NL63
Source: Front Cell Infect Microbiol. 2024 Apr 26;14:1378804. doi: 10.3389/fcimb.2024.1378804 (PMC11082418; doi:10.3389/fcimb.2024.1378804)
Supplement: Supplementary file 3 [file Image_3.pdf]

S3

Diagnosis profile of patients infected with HCoV-NL63 subgenotype C3 C4 and B

**Diagnosis**	**Case number**		
	**C3**	**C4**	***P*-value[a]**
acute bronchitis	1	1	0.517
acute pharyngitis	3	8	1.000
acute tonsillitis	2	6	1.000
acute upper respiratory tract infection	3	4	0.706
acute laryngitis	0	2	-
acute gastroenteritis	1	0	-
	**C3**	**B**	***P*-value[a]**
acute pharyngitis	3	1	0.771
acute tonsillitis	2	2	1.000
acute upper respiratory tract infection	3	4	0.657
	**C4**	**B**	**P-value[a]**
acute pharyngitis	8	1	0.483
acute tonsillitis	6	2	1.000
acute upper respiratory tract infection	4	4	0.147

[a] Fisher’s exact T-test
