# Supplementary material for: Seasonal human coronavirus NL63 epidemics in children in Guilin, China, reveal the emergence of a new subgenotype of HCoV-NL63
Source: Front Cell Infect Microbiol. 2024 Apr 26;14:1378804. doi: 10.3389/fcimb.2024.1378804 (PMC11082418; doi:10.3389/fcimb.2024.1378804)
Supplement: Supplementary file 4 [file Image_4.pdf]

S4

Positive rate and Proportion of 15 respiratory viruses in 365 ARIs case

Virus	Positive case	Positive Rate (%)	Proportion (%)
Single infection	284	44.51	77.81
FluA	54	8.46	14.79
FluB	15	2.35	4.11
RSV	13	2.04	3.56
HRV	44	6.90	12.05
AdV	36	5.64	9.86
HBoV	21	3.29	5.75
NL63	45	7.05	12.32
OC43	6	0.94	1.64
229E	0	0	0
HKU1	0	0	0
B19	1	0.16	0.27
PIV1	13	2.03	3.56
PIV2	6	0.94	1.64
PIV3	0	0	0
hMPV	30	4.70	8.22
Double infections	70	10.97	19.18
HRV+FluA	3	0.47	0.82
HRV+FluB	4	0.63	1.10
FluA+FluB	1	0.16	0.27
RSV+ FluB	1	0.16	0.27
AdV+HRV	2	0.31	0.55
AdV+FluA	2	0.31	0.55
AdV+RSV	3	0.47	0.82
AdV+FluB	1	0.16	0.27
NL63+RSV	15	2.35	4.11
NL63+AdV	7	1.10	1.92
NL63+HBOV	6	0.94	1.64
NL63+HRV	3	0.47	0.82
HBOV+FluA	6	0.94	1.64
HBOV+RSV	3	0.47	0.82
HBOV+HRV	5	0.78	1.37
HBOV+AdV	2	0.31	0.55
OC43+HRV	1	0.16	0.27
OC43+FluB	1	0.16	0.27
0C43+HBOV	1	0.16	0.27
PIV2+hMPV	1	0.16	0.27
hMPV+HRV	1	0.16	0.27
hMPV+RSV	1	0.16	0.27
Multiple infections	12	1.88	3.29
NL63+AdV+RSV	3	0.47	0.82
NL63+HBOV+FluA	1	0.16	0.27
NL63+HRV+FluA	2	0.31	0.55

HBOV+RSV+FluA	1	0.16	0.27
HBOV+AdV+FluB	2	0.31	0.55
HBOV+AdV+FluA	1	0.16	0.27
hMPV+HBOV+AdV	1	0.16	0.27
NL63+AdV+RSV+FluB	1	0.16	0.27
